# The effect of prior compression tests on the plantar soft tissue compressive and shear elastic properties

**DOI:** 10.1186/1757-1146-5-S1-P14

**Published:** 2012-04-10

**Authors:** Shruti Pai, Paul T Vawter, William R Ledoux

**Affiliations:** 1 VA RR&D Center of Excellence for Limb Loss Prevention and Prosthetic Engineering, Seattle, Washington, 98108, USA; 2 Mechanical Engineering, University of Washington, Seattle, Washington, 98195, USA; 3 Orthopaedics and Sports Medicine, University of Washington, Seattle, Washington, 98195, USA

## Background

Changes in the shear plantar soft tissue properties with diabetes likely play a role in plantar ulceration, yet little is known about these characteristics. We recently conducted *in vitro* shear tests on specimens previously tested in compression to characterize the tissue under both these loading modes. However, previously tested specimens might not provide representative mechanical properties as prior testing may have altered the tissue. The purpose of this study was to test the effect of prior compression testing on the plantar soft tissue shear and compressive properties using paired specimens in a two-part study.

## Materials and methods

Four pairs of cylindrical specimens (n=8) were isolated per previous methods [1] from the calcaneus and lateral midfoot from three fresh-frozen, non-diabetic older cadaveric donors. In the first part of the study, one specimen from each pair was subject to compressive loading with modifications to compare properties before and after testing. In the second part, both paired specimens were subject to shear loading, i.e., both the previously compression tested from the first part and the previously untested specimens.

## Results

The results (Table 1) of the first part demonstrated that prior compression testing affects the plantar soft tissue compressive properties by reducing peak stress and modulus by two to three times, although additional testing is needed since these results were likely confounded by stress softening effects. In contrast, in Part B, none of the elastic shear properties were affected by prior testing in compression.

**Table 1:** Mean [SE] nonlinear elastic compressive and shear data parameters

	U	C	p*
Peak compressive strain (%)	39.99 [3.6e-3]	39.98 [6.7e-3]	0.3
Peak compressive stress (kPa)	31.6 [6.3]	12.9 [4.5]	0.0002
Compressive modulus (kPa)	267 [72]	87 [35]	0.0031
Compressive energy loss (%)	38.2 [1.4]	37.8 [1.6]	0.7
			
Peak shear strain (%)	80.9 [1.6e-2]	80.9 [1.6e-2]	0.3
Peak shear stress (kPa)	9.0 [1.5]	9.9 [2.0]	0.6
Initial shear modulus (kPa)	58 [19]	61 [16]	0.8
Toe shear modulus (kPa)	5.4 [0.7]	5.3 [0.9]	0.9
Final shear modulus (kPa)	21.9 [4.0]	25.4 [5.6]	0.3
Shear energy loss (%)	48.0 [4.2]	42.8 [4.2]	0.2

p<0.05 indicates significance for linear mixed effects regression; U = previously untested, C = compression tested.

## Conclusions

This study demonstrates that prior compression testing of the plantar soft tissue may alter the compressive properties. However, since the shear parameters were not affected by prior testing in compression, shear tests using previously compression tested specimens should provided representative properties.
